# Proteins and Minerals in Whey Protein Supplements

**DOI:** 10.3390/foods12112238

**Published:** 2023-06-01

**Authors:** Dailos González-Weller, Soraya Paz-Montelongo, Elena Bethencourt-Barbuzano, Daniel Niebla-Canelo, Samuel Alejandro-Vega, Ángel J. Gutiérrez, Arturo Hardisson, Conrado Carrascosa, Carmen Rubio

**Affiliations:** 1 Health Inspection and Laboratory Service, Servicio Canario de la Salud, 38006 Santa Cruz de Tenerife, Spain; 2 Toxicology Department, Universidad de La Laguna, 38071 La Laguna, Spain; 3 Department of Animal Pathology and Production, Bromatology and Food Technology, Faculty of Veterinary, Universidad de Las Palmas de Gran Canaria, 35413 Arucas, Spain

**Keywords:** protein supplements, whey protein, proteins, chemical elements, potentially toxic elements

## Abstract

Sports nutrition supplementation is a widespread practice. Whey protein supplements contribute not only to protein intake but also to dietary exposure to minerals. The labelling present provides the percentage of protein and rarely refers to other components, such as potentially toxic elements such as B, Cu, Mo, Zn, and V that present tolerable upper intake levels set by the European Food Safety Authority. The percentage of protein declared on supplement labelling was checked using the Kjeldahl method, and the levels of Ca, Mg, K, Na, Ba, B, Co, Cu, Cr, Sr, Fe, Li, Mn, Mo, Ni, V, Zn, and Al were analyzed by ICP-OES with the aim of characterizing the protein and mineral contents of isolate and concentrate whey protein supplements representative of the European market. The protein content was 70.9% (18–92.3%) and statistically significant differences were observed between the declared and real protein percentages. Among the minerals, K (4689.10 mg/kg) and Ca (3811.27 mg/kg) presented the highest levels, whereas Co (0.07 mg/kg) and V (0.04 mg/kg) showed the lowest levels. It was concluded that the quality and safety of these products needs to be monitored and regulated. A high degree of non-compliance with labelling claims was detected. Furthermore, the contributions to the recommended and tolerable intakes among regular consumers need to be assessed.

## 1. Introduction

Nutrition and hydration fundamentally influence an athlete’s health and performance. Therefore, in addition to various factors such as training, motivation, and the absence of injuries, among others, an appropriate choice of foods and drinks, in combination with an organized nutritional plan, is crucial for physical exercise to reach its full potential [1,2]. It is becoming more and more common to use ergogenic nutritional aids and supplements to improve and increase physical performance and minimize the manifestations of fatigue without endangering the health of the athlete or violating the sporting spirit [2,3]. These ergogenic nutritional aids can be differentiated into nutritional modifications to the specific diet of the athlete (based on changes in the amounts or contributions of the usual dietary components such as carbohydrates, fatty acids, branched-chain amino acids, and vitamins, among others) and nutritional/dietary supplements (products that provide special nutritional requirements both for exercise and to prevent or reverse nutritional deficiencies) [3].

As a result, sports nutrition supplementation is becoming increasingly widespread, with protein supplements being one of the most widely used [4,5,6]. The benefits attributed to them make them very attractive to both professional and amateur athletes [7,8].

The protein in these supplements can be obtained from whey casein, egg, and vegetable protein, with the quality and speed of absorption varying according to their origin [2]. It should also be noted that the source of protein used may vary from one batch to another, as whey is influenced by the animal’s diet and environmental factors, among others. The most used supplements are made from whey protein, which is a mixture of globulins and caseins contained in whey from cheese production. This protein is of high quality, as it is rich in essential branched-chain amino acids and is also rapidly absorbed. Due to its high nutritional value and the fact that it promotes the growth and maintenance of muscle mass, the use of whey protein as a nutritional supplement has increased significantly in recent years [9,10].

However, one of the problems associated with the use of these types of supplements lies not only in their incorrect use or abuse because of how they are consumed ignoring the real functions and purposes of the product [11] but also in the potential fraudulent information provided [12,13,14] such as the presence of lower or higher amounts of nutrients and active components than those stated on the labelling or a lack of information on the composition, among others [9,10,13,14].

It has been estimated that more than 400 substances other than vitamins and minerals are used in the composition of food supplements. In addition to proteins, these novel products may contain other active ingredients such as Na, Mg, Ca, Mo, Ni, K, Sr, B, V, Zn, and Al, some of which may be considered potentially toxic elements (PTEs) such as B, Cu, Mo, Zn, and V. For them, the EFSA (European Food Safety Authority) has established a tolerable upper intake level (uL) of 0.16 mg B/kg b.w./day; 5 mg Cu/day; 0.6 mg Mo/day, 25 mg Zn/day, and 0.026 mg V/day. The UL indicates the maximum amount that can be consumed without implying a risk to health [15]. The intake of high amounts of protein supplements may expose regular consumers to these PTEs, generating potential health risks.

Although the use of protein supplements may be very useful for certain consumers, this consumption should occur under the supervision of a healthcare provider since unsupervised self-consumption may carry several negative outcomes [16]. Among the most noteworthy risks associated with protein supplements, alterations in renal function [17,18], effects on the gut microbiota [19], and the development of acne stand out [20].

Despite the recommendations of organizations such as the EFSA and the IOC (International Olympic Committee), food supplements do not have a specific and harmonized European legal framework and are still currently regulated as foodstuffs. Therefore, sports nutrition supplements in the European Union (EU) are regulated by each country’s regulations on food supplements or medicines [1]. Furthermore, it is common to observe the marketing and commercialization of products that do not comply with EU labelling requirements. Moreover, cases of fraud continue to be reported [12,14].

In view of the above, the objectives of this work were to determine protein richness and compliance with the labelling of several whey protein supplements commercialized in Europe and to quantify the levels of calcium (Ca), magnesium (Mg), potassium (K), sodium (Na), barium (Ba), boron (B), cobalt (Co), copper (Cu), chromium (Cr), strontium (Sr), iron (Fe), lithium (Li), manganese (Mn), molybdenum (Mo), nickel (Ni), vanadium (V) zinc (Zn), and aluminum (Al) to characterize the mineral profile of these novel foods.

## 2. Materials and Methods

A total of 47 samples of whey protein supplements purchased from different points of sale (online shops (8 samples), gyms (7 samples), pharmacies (13 samples), sports shops (9 samples), supermarkets and hypermarkets (1 sample), and specialized shops (9 samples)) between March 2021 and April 2022 were analyzed. All samples were produced in the EU except six which were from the United Kingdom. The labelling of the packages was studied, and the protein content was recorded. Twenty-two of these forty-seven samples were protein supplements concentrate and twenty-one were protein supplements isolate. The samples (0.2–0.5 g for protein analysis and 5 g for elemental analysis) were stored in polyethylene jars and kept at room temperature for further processing in less than 1 month.

1. Determination of total protein in protein supplements

Although the determination of total protein by spectrophotometric methods is commonly used in several areas, the Kjeldahl method [21,22] is still the officially recognized standard reference method for the determination of protein content in foods. The Kjeldahl method is based on determination of the organic nitrogen concentration and three main steps: digestion, distillation, and titration [23,24,25,26]:

- Digestion of samples (0.3–0.5 g) by addition of H_2_SO_4_ (98% concentration) (10 mL) and two catalyst tablets (Cu-Se) (Kjeldahl Catalyst Cu-Se, 1.5% CuSO_4_. 5H_2_O + 2%Se. Tablets. Panreac, AppliChem, Barcelona Spain) in a digester at a range of 350–380 °C for 1 h [25,26]. The organic nitrogen is converted to NH_4_^+^ under these conditions.
- Distillation of the digested solution using steam and a Kjeldahl distiller (FOSS KT 200 Kjeltec™ nitrogen distiller). Before starting the distillation, the flasks were prepared and ten drops of Mixed Indicator 5 (Methyl Red-Bromocresol Green, 283303, Panreac, AppliChem, Barcelona, Spain) and 30 mL of saturated H_3_BO_3_ were added, producing a fuchsia coloring with pH 4–5.5. The NH_3_^+^ was distilled and collected in a receiver vessel.
- Evaluation of the distillation results by addition of HCl (0.1000 mol/l) (181,023.1214, Panreac, AppliChem, Barcelona, Spain) with an electronic burette until the color changes from green to fuchsia with five drops of methyl red indicator to 0.1% (281,618.1208, Panreac, AppliChem, Barcelona, Spain). The green color is produced at pH 4.2–6.2, thus determining the protein nitrogen.

The calculation of the percentage of total protein from whey is obtained as follows. The percentage of nitrogen through the H_3_BO_3_ solution that acts as a receiving solution for total ammonia is calculated according to Equation (1) below:

% Nitrogen = (ml measurement acid − ml blank) × N of acid × 1.007/sample weigh (g)(1)

Considering this percentage of total nitrogen, the protein percentage is calculated by considering a conversion factor, which in the case of protein supplements of animal origin (milk, cheese, milk powder, and milk products) is set at 6.38 according to Equation (2) below [25,27,28]:

% Total Protein = % Nitrogen × Protein Factor(2)

The analysis of each sample was carried out in triplicate and the final results for each sample are the corresponding means. The data are summarized as the medians and interquartile ranges (25th, 50th, and 75th percentiles) for the statistical study and the data referenced on the package labels. Distributions were compared using the Shapiro–Wilk test (*p* < 0.05) for paired data. The Mann–Whitney test was used to determine the independent variables. A hypothesis test was considered statistically significant when the corresponding *p*-value was less than 0.05. The data were analyzed using R package, version 4.2.2 [29].

2. Element analysis in protein supplements

Approximately 5 g of whey protein supplements sample was weighed in triplicate and dried in an oven (60–80 °C) for 12–14 h before being transferred to a muffle furnace for incineration. The temperature (T) was gradually raised (approximately 50 °C every hour) until reaching 425 ± 15 °C. This T was maintained for 48 h. The white ashes obtained were dissolved in 1.5% HNO_3_.

The element analysis was performed by inductively coupled plasma optical emission spectrometry (ICP-OES) as it is not only the reference technique but it also presents high sensitivity and reproducibility [30,31,32].

Certified standard solutions were used for the calibration curves. While the certified standard IV-STOCK-2 from Inorganic Ventures was used for the minerals Ca, Mg, K, and Na, the certified standard multi-element std SCP28AES from SCP Science was used for the rest of the minerals (Co, Cu, Cr, Fe, Mn, Mo, Ni, V, Zn, Ba, B, Sr, Li, and Al). Instrumental limits of detection and quantification were estimated by analyzing fifteen targets under reproducibility conditions [33]. The operating parameters, limits of detection and quantification, as well as the wavelengths of each mineral are shown in Table A1.

The required accuracy and coefficient of variation values were set at a maximum of 10%, with lower values being obtained for all minerals in the study. The reference materials SRM Oyster Tissue 1566b, SRM 1573a Tomato Leaves, and SRM 1515 Apple Leaves were used. Two replicates of each sample were analyzed, and a mean concentration and a % RSD value for the quantifiable ranges of the method were obtained from each replicate. A % RSD ≤ 10% was set to determine a measurement as valid.

Prior to sample preparation, all of the materials used were washed with a laboratory cleaning detergent to prevent contamination and to remove possible traces of minerals and kept in 5% HNO_3_ for 24 h followed by washing with milli-Q quality water.

3. Statistical analysis

The protein content statistical analysis was realized by the Jamovi Project (2021) (Version 2.2) R Core Team Language (2021) (Version 4.0) [34]. Statistical analysis of the minerals was performed using GraphPad Prism 8.0.1. software (GraphPad, San Diego, CA, USA). The distribution of the results was studied by applying the following normality tests: Anderson–Darling, D’Agostino and Pearson, Shapiro–Wilk, and Kolmogorov–Smirnov [35]. As the results did not follow a normal distribution, Mann–Whitney non-parametric tests were used [36,37]. A value of *p* < 0.05 was considered a significant difference. In addition, a study was carried out to obtain confidence intervals (95%) [38].

## 3. Results and Discussion

1. Protein richness of whey protein supplements and discrepancies with labelling

**Figure 1:**
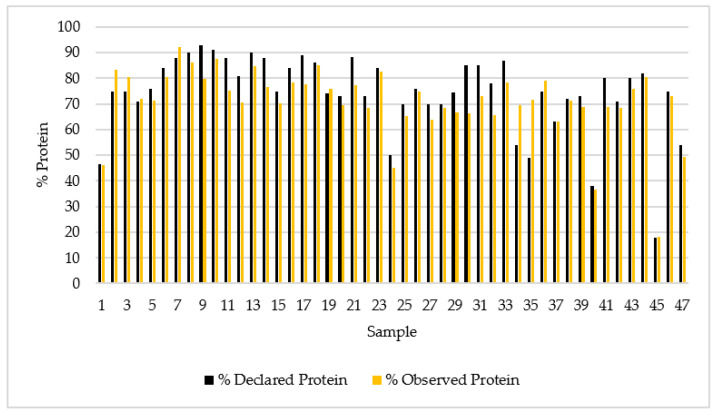
Protein percentage declared and observed in the 47 whey protein supplements.

Figure 1 shows the protein percentages obtained in the present study and those declared in the nutritional analysis of the dietary supplements sampled.

The results obtained from the statistical analysis show that the mean average value of the declared protein content was 74.3% and the calculated protein content was 70.9%. Specifically, eight samples (17% of the total) had a protein percentage slightly higher than that declared on the label. This highlights the need to enforce the monitoring and regulation of sports supplements. The statistical results showed standard deviation values of 15.2 and 13.5 for the reported and observed protein percentages, respectively. The *p*-value for the Mann–Whitney test was 0.04 which makes both variables independent (Table 1).

These values are similar to those found in the review published by Martínez-Sanz et al. [13], where the results of several studies on nutritional supplements were documented [39,40]. Schönfeldt et al. [39] studied the composition of seventy protein powder samples from South Africa and their discussion of the results was similar to the one performed in the present paper, finding significant differences between the actual protein content and that declared in the labelling (30% of the samples deviated by 10% from the declared protein and the remaining 70% deviated by 5%). In the other study, Garrido et al. [40] reported that a 37% of nutritional supplement samples analysed contained vegetal proteins, when they should have only contained whey protein. Pellegrino et al. [41] described that the origin protein (composition) should also be considered in the final product quality.

As previous studies have demonstrated, lower amounts of protein were found than those in the nutrition labelling of the products tested. It is therefore clear that, despite slight changes in the legislation on nutritional supplements, there is still no comprehensive control to ensure quality. The nutritional information of most protein supplements analyzed does not comply with the national regulation on food supplements (Spanish Royal Decree 130/2018).

2. Minerals in whey protein supplements

The number of samples analyzed, the concentrations obtained for each of the minerals determined (mean ± SD) (wet weight), as well as the maximum and minimum values and the number of samples with concentrations above the limit of quantification for each element are shown in Table 2. Ca, Mg, K, and Na are the minerals with the highest concentrations, and V and Co were characterized as the PTEs with the lowest mean concentrations.

It should be noted that the variability in the results is high for some minerals. However, this is considered normal, as the content of minerals in foods depends on a variety of factors, ranging from production and processing methods to the environmental conditions of the whey origin [42]. The high levels of some minerals, besides being related to their biological origin, could also be justified because some salts are used as additives in these whey protein supplements. Thus, for example, sodium hydro phosphate is used as a flavor enhancer, potassium hydro phosphate and potassium citrate are used as pH buffers, sodium and potassium chlorides are used as electrolytes, and calcium phosphate is as an anti-caking agent [10].

The large confidence intervals obtained in most cases (Table A2) highlight the lack of homogeneity of the samples analyzed even when sampling included just animal protein supplements from whey and no other sources. This fact is noteworthy since the sampling was designed to have a low diversity and to show homogeneity.

**Figure 2:**
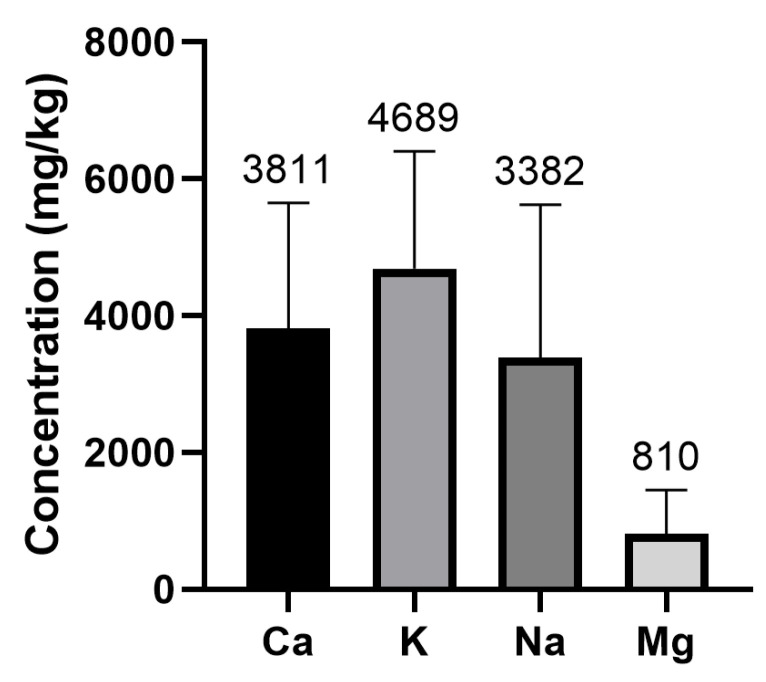
Minerals contents (mg/Kg) in whey protein supplements.

The results show that whey protein supplements are a source of minerals. In the case of Na, Mg, Ca, and K, the levels in these products are high, and protein supplements could be considered relevant dietary sources of these nutritional elements. While the mineral with the highest concentration is K (mean average concentration: 4689.10 mg/kg), the mineral with the lowest concentration is Mg (810 mg/kg) (Figure 2). Significant differences were detected between K vs. Ca (*p* = 0.0013), Ca vs. Mg (*p* < 0.0001), K vs. Na (*p* < 0.0001), K vs. Mg (*p* < 0.0001), and Na vs. Mg (*p* < 0.0001). However, no significant differences were detected between Ca vs. Na content (*p* = 0.0806).

**Figure 3:**
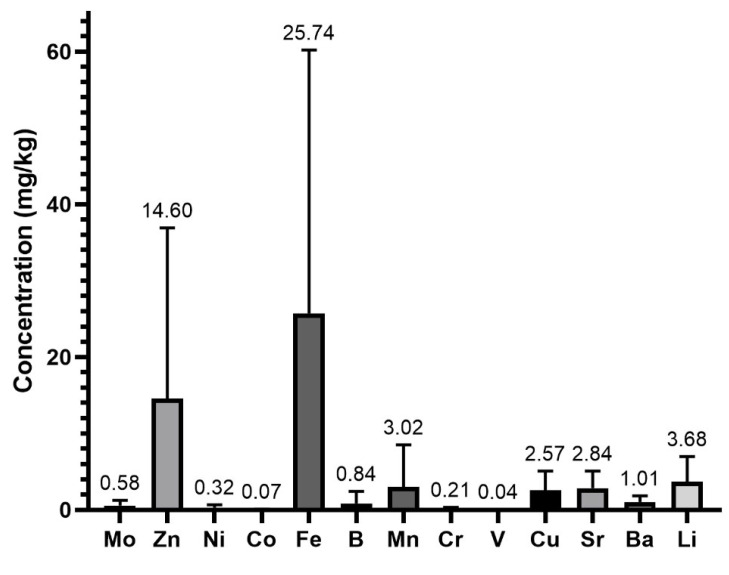
Microelements and PTE content (mg/Kg) in the protein supplements.

The microminerals that were quantified in the highest concentrations were Fe and Zn with concentrations of 25.74 and 14.60 mg/kg, respectively (Figure 3). Co, with levels of 0.07 mg/kg and V, with levels of 0.04 mg/kg, were the minerals with the lowest concentrations of this group of minerals. Significant differences were detected in the content of all trace elements, except for Mo vs. B (*p* = 0.1808), Ni vs. Cr (*p* = 0.9520), Co vs. V (*p* = 0.0873), and Ni vs. Cr (*p* = 0.9520). These differences may be due to the different ingredients and procedures that may have been applied by the manufacturers of these products.

For some of the PTEs analyzed, such as Mo, even the low levels detected are toxicologically relevant since the contribution of regular consumption of these whey protein supplements to the upper intake level (UL) of 0.6 mg Mo/day set by EFSA [15] could be high and become a dietary hazard that may need to follow a risk characterization. In the case of B and V, their levels in the whey protein supplements are low. Therefore, even if large amounts are consumed daily, the intake of these PTEs from this dietary source will hardly become a dietary hazard. In the case of the only non-essential element analyzed in the present study (Al), its mean concentration was found to be 7.19 mg/kg.

Because different types of whey protein supplements are found on the market (isolate and concentrate), the differences in the minerals’ occurrence were also investigated (Table 3). In the case of Na, K, Mg, and Ca, no major differences were observed if the mean concentrations are considered, although for all of them, the levels are higher in the isolate protein supplements. The differences are noticeable if the maximum values detected are considered, especially in Ca, which rises from 4729.49 mg/kg for the concentrate whey protein supplements to 11,000.50 for the isolate whey protein supplements. Nevertheless, the choice of one type or another of protein supplements will not involve large variations in terms of the contributions to the recommended intakes (Table 3).

Unlike what happens for Na, K, Mg, and Ca, some of the PTEs (Mo, Cu, Co, Ba, B, V, and Al) present higher values in those concentrate protein supplements. The case of Mo is striking since its levels in concentrate products (0.82 mg/kg) are three times the levels detected in isolate protein supplements (0.28 mg/kg). In the case of Zn, something similar to Mo occurs. Concentrate protein supplements show Mo average levels of 10 mg/kg, almost double the levels observed in isolate protein supplements. For B, the opposite is observed. The highest B concentration was observed in the concentrate whey protein supplements (Table 3).

Table 4 shows a comparison with previous studies [5,6,9,10,11,18]. As can be observed, this study is the first one to study and report B levels in protein supplements. When comparing the concentrations of the macrominerals from this study with those of other previously published studies, the content of Ca (3811.27 mg/kg) is notably like that reported by Elgammal et al. (4423.68 mg/kg) and higher than that of Guefai et al. For the rest of the macrominerals, the levels of Mg, K, and Na were fairly similar to those reported by Elgammal et al. and Guefai et al. [5,6].

The observed Ba levels were higher than those described by Guefai et al. but similar to those of Pinto et al. [6,18]. The concentration of Co was very similar to that of all the studies consulted. Regarding Cu, its levels were slightly higher than those of Pinto et al. and Lofaso but lower than those of Guefai et al. [6,11,18]. Cr, Mn, and Zn were within the concentration ranges reported by the studies consulted. The mean Fe concentration was higher than that of all previously published studies, although like that found by Guefai et al. [6]. The levels of Ni and Sr coincided and were notably similar to those of the other authors in studies where these elements were quantified (Table 4).

Finally, the concentration of Mo was lower than that reported in the only study found in the literature where Mo was analyzed [18], and that of V was slightly higher than that of Pinto et al. and Lofaso and practically the same as that of Guefai et al. [6,11,18]. In the case of Al, the mean concentration observed (7.19 mg/Kg) is within the range of concentrations of the study consulted [18], and it was notably like, although slightly lower than, that reported by Guefai et al. [6] (Table 4).

## 4. Conclusions

The growth of dietary supplementation makes protein supplements attractive products for both the food and pharmaceutical industries and consumers. Protein supplements have experienced exponential diversification and marketing, and consumer profiles are continuously changing as the situations and contexts of consumption of these nutritional supplements are broadened.

Protein supplements are not only rich in proteins but are also a source of elements of nutritional interest and PTEs such as Al, B, Cu, Mo, Ni, Zn, and V. This is the first published study to observe the occurrence of boron in protein supplements. Protein supplements should be considered as relevant dietary sources of minerals among regular consumers.

We believe the contributions of the daily consumption of these products to the total intakes of these PTEs should be assessed in total diet exposure studies and risk characterization analysis with the aim of preventing the health risks associated. Furthermore, protein supplements’ quality and safety should be assessed and monitored as not only have discrepancies in the labeled protein percentage been detected but also as some PTEs with limited dietary intakes, such as Mo and Cr, have been observed in considerable concentrations.

## Figures and Tables

**Table 1:** Statistical results of the proteins analyzed and labeled in whey protein supplements.

	Declared	Observed
Samples (N)	47	47
Mean	74.3%	70.9%
Std. error mean	2.22	1.96
Median	75.0%	71.9%
Mode	75.0%	71.3%
Sum	3493	3334
Standard deviation	15.2	13.5
Minimum	18%	18.2%
Maximum	93.0%	92.3%
Shapiro–Wilk W	0.849	0.842
Shapiro–Wilk p	<0.001	<0.001
25th percentile	71.0%	71.9%
50th percentile	75.0%	71.9%
75th percentile	85.0%	78.8%

**Table 2:** Minerals (g/kg and mg/Kg wet weight) in whey protein supplements.

Minerals	N	N (>LQ)	Mean Concentration ± SD (g/Kg)	Maximum Value (g/Kg)	Minimum Value (g/Kg)
Ca	47	47	3.81 ± 1.84	11.00	0.48
Mg	47	47	0.81 ± 0.64	3.25	0.06
K	47	47	4.70 ± 1.71	9.69	0.24
Na	47	47	3.38 ± 2.24	11.42	0.24
**Minerals**	**N**	**N (>LQ)**	**Mean Concentration ± SD** **(mg/Kg)**	**Maximum Value** **(mg/Kg)**	**Minimum Value** **(mg/Kg)**
Ba	47	47	1.01 ± 0.84	5.05	0.23
B *	47	43	0.84 ± 1.58	10.56	<LQ
Co	47	28	0.07 ± 0.06	0.31	<LQ
Cu *	47	47	2.57 ± 2.51	10.42	0.38
Cr	47	47	0.21 ± 0.14	0.60	0.03
Fe	47	47	25.74 ± 34.45	175.64	2.19
Li	47	46	3.68 ± 3.31	14.11	<LQ
Mn	47	47	3.02 ± 5.49	26.87	0.09
Mo *	47	47	0.58 ± 0.68	4.26	0.05
Ni	47	47	0.32 ± 0.34	1.40	0.01
Sr	47	47	2.84 ± 2.22	10.36	0.37
V *	47	22	0.04 ± 0.03	0.14	<LQ
Zn *	47	47	14.60 ± 22.23	101.51	1.35
Al	47	47	7.19 ± 7.37	35.22	0.40

N: number of samples analyzed. N (>LQ): number of samples whose concentration is > LQ. * potentially toxic elements (PTE) for which the EFSA has established a tolerable upper intake level (UL) of 0.16 mg B/kg b.w./day; 5 mg Cu/day; 0.6 mg Mo/day; 25 mg Zn/day; and 0.026 mg V/day [15].

**Table 3:** Differences in the minerals’ contents between the concentrate and isolate whey protein supplements.

Whey Protein Supplement Concentrate				Whey Protein Supplement Isolate			
Mineral	N	N (>LQ)	Mean Concentration ± SD (g/kg) (C_min_–C_max_)	Mineral	N	N (>LQ)	Mean Concentration ± SD (g/kg) (C_min_–C_max_)
Ca	22	22	3.17 ± 1.14 (0.48–4.73)	Ca	21	21	4.59 ± 2.18 (2.31–11.43)
Mg	22	22	0.70 ± 0.54 (0.56–2.69)	Mg	21	21	0.96 ± 0.77 (0.36–3.25)
K	22	22	4.48 ± 1.57 (0.24–7.13)	K	21	21	5.11 ± 1.91 (1.62–9.70)
Na	22	22	3.22 ± 1.89 (0.24–7.19)	Na	21	21	3.53 ± 2.66 (0.69–11.43)
**Mineral**	**N**	**N (>LQ)**	**Mean Concentration ± SD (mg/kg)** **(C_min_–C_max_)**	**Mineral**	**N**	**N (>LQ)**	**Mean Concentration ± SD (mg/kg)** **(C_min_–C_max_)**
Ba	22	22	1.06 ± 1.11 (0.23–5.05)	Ba	21	21	0.92 ± 0.42 (0.40–2.05)
B *	22	19	1.18 ± 2.33 (0.17–10.56)	B	21	21	0.54 ± 0.39 (0.15–1.65)
Co	22	11	0.08 ± 0.08 (0.03–0.31)	Co	21	14	0.05 ± 0.02 (0.02–0.10)
Cu *	22	22	2.65 ± 2.66 (0.38–10.42)	Cu	21	21	2.40 ± 2.42 (0.49–9.50)
Cr	22	22	0.19 ± 0.14 (0.03–0.59)	Cr	21	21	0.21 ± 0.12 (0.05–0.46)
Fe	22	22	20.18 ± 36.75 (2.19–175.64)	Fe	21	21	26.94 ± 24.27 (3.96–74.71)
Li	22	21	3.61 ± 3.70 (0.35–13.84)	Li	21	21	3.75 ± 3.29 (0.51–14.11)
Mn	22	22	2.41 ± 4.87 (0.09–19.74)	Mn	21	21	3.70 ± 6.45 (0.23–26.87)
Mo *	22	22	0.82 ± 0.86 (0.05–4.26)	Mo	21	21	0.28 ± 0.27 (0.06–1.04)
Ni	22	22	0.31 ± 0.38 (0.02–1.40)	Ni	21	21	0.32 ± 0.31 (0.03–1.13)
Sr	22	22	2.44 ± 2.13 (0.37–10.36)	Sr	21	21	3.02 ± 1.97 (1.37–10.01)
V *	22	8	0.05 ± 0.04 (0.02–0.14)	V	21	11	0.04 ± 0.02 (0.02–0.08)
Zn *	22	22	10.00 ± 13.94 (1.35–55.0)	Zn	21	21	18.63 ± 28.67 (1.36–101.51)
Al	22	22	7.70 ± 9.30 (0.42–35.22)	Al	21	21	6.05 ± 3.91 (1.82–13.46)

N: number of samples analyzed. N (>LQ): number of samples whose concentration >LQ. * Potentially toxic elements (PTEs) for which the EFSA has established a tolerable upper intake level (UL) of 0.16 mg B/kg b.w./day; 5 mg Cu/day; 0.6 mg Mo/day; 25 mg Zn/day; and 0.026 mg V/day [15].

**Table 4:** Comparison with previous studies on minerals in protein supplements (mean concentrations, mg/kg).

	Elgammal et al. [5]	Guefai et al. [6]	Lofaso et al. [11]	Pinto et al. [18]	This Study
**Ca**	4423	1064	-	-	3811
**Mg**	962	724	-	-	809
**K**	-	4978	-	-	4689
**Na**	2830	3575	-	-	3382
**Ba**	-	0.46	-	1.38	1.01
**B**	-	-	-	-	0.84
**Co**	N.D.–< 0.5	0.35	0.01	0.04	0.07
**Cu**	N.D.–16.78	4.00	1.91	1.90	2.57
**Cr**	N.D.–0.69	1.40	0.05	0.46	0.21
**Fe**	17.51	22.00	11.5	13.70	25.74
**Li**	-	1.19	-	0.02	3.68
**Mn**	3.56	8.70	0.20	1.60	3.02
**Mo**	-	-	-	0.92	0.58
**Ni**	N.D.–0.93	0.52	-	0.35	0.32
**Sr**	-	3.10	-	3.20	2.84
**V**	-	0.44	0.002	0.025	0.04
**Zn**	17.66	29.10	-	6.70	14.60
**Al**	<5–16.26	8.00	-	3.00	7.19

N.D.: Not detectable.

## Data Availability

The data presented in this study are available on request from the corresponding author.

## Appendix A

**Table A1:** Operating parameters, limits of detection, quantification, and wavelength of each mineral determined by ICP-OES. ICP-OES operating parameters.

Mineral	Emission Wavelengths (nm)	Detection Limits (mg/L)	Quantification Limits (mg/L)
Al	167.0	0.005	0.015
B	249.6	0.008	0.027
Ba	455.4	0.0006	0.002
Ca	315.8	1.629	5.432
Co	228.6	0.001	0.005
Cr	267.7	0.001	0.005
Cu	324.7	0.003	0.011
Fe	238.2	0.004	0.013
K	766.4	1.764	5.883
Li	670.7	0.013	0.031
Mg	383.8	1.580	5.268
Mn	257.6	0.0008	0.003
Mo	202.0	0.0016	0.005
Na	818.3	2.221	7.404
Ni	221.6	0.0009	0.003
Sr	407.7	0.003	0.011
V	292.4	0.0014	0.004
Zn	213.8	0.0027	0.009

RF power 1150 W; nebulizer gas flow 12.5 L/min; cool gas flow 12.5 L/min; nebulizer gas pressure 0.2 L/min; auxiliary gas flow 0.5 L/min; pump speed 45 rpm.

**Table A2:** Confidence intervals considering a confidence level of 95%**.** Confidence intervals considering a confidence level of 95%.

	Lower 95% CI of the Mean	Upper 95% CI of the Mean
Protein (%)	17.43	33.79
Mineral		
Ca	1875	2966
Mg	365.1	663.3
K	2364	3592
Na	1587	2709
Al	2.988	6.144
B	0.1957	0.7835
Ba	0.4511	0.8357
Co	0.01324	0.03487
Cr	0.09931	0.1704
Cu	1.086	2.175
Fe	9.383	23.31
Mo	0.2304	0.5115
Mn	0.8539	2.985
Ni	0.1296	0.2744
Sr	1.288	2.324
V	0.007289	0.01866
Zn	4.851	13.69
